# Reduction of Serum Concentrations and Synergy between Retinol, β-Carotene, and Zinc According to Cancer Staging and Different Treatment Modalities Prior to Radiation Therapy in Women with Breast Cancer

**DOI:** 10.3390/nu11122953

**Published:** 2019-12-04

**Authors:** Cintia Rosa, Carlos Franca, Sérgio Lanes Vieira, Antônio Carvalho, Antônio Penna, Carla Nogueira, Suzane Lessa, Andrea Ramalho

**Affiliations:** 1 Social Applied Nutrition Department, Micronutrients Research Center (NPqM), Federal University of Rio de Janeiro, Rio de Janeiro 21.941-902, Brazil; cfendlich@gmail.com (C.F.); carlafrancanut@yahoo.com.br (C.N.); suzane.laura@gmail.com (S.L.); aramalho.rj@gmail.com (A.R.); 2 Department of Radiation Oncology, Ingá Radiotherapy Clinic (CRI), Brazilian Institute of Oncology (IBO), Federal University of Rio de Janeiro, Niterói 24.210-470, Brazil; cringa@cringa.com.br (S.L.V.); antoniopcringa@cringa.com.br (A.P.); 3 Department of Radiation Oncology, Pontifical Catholic University of Rio de Janeiro, Rio de Janeiro 22.451-900, Brazil; 4 Department of Radiology, Clementino Fraga Filho University Hospital, Federal University of Rio de Janeiro, Rio de Janeiro 21.941-902, Brazil; antoniocars@gmail.com

**Keywords:** radiotherapy, antioxidants, toxicity, chemotherapy, conservative surgery, breast cancer

## Abstract

The procedures used for breast cancer treatment are able to increase the level of oxidative stress and cause depletion of antioxidants. Objectives: To investigate the relationship between serum concentrations of retinol, β-carotene, and zinc, according to breast cancer staging, considering different treatment modalities prior to radiation therapy and the synergistic action between these micronutrients. Methods: This is a cross-sectional observational study comprising a cohort of patients with breast cancer which was carried out prior to radiation therapy. Patients were divided into 3 groups: G1 comprised women who had undergone breast-conserving surgery, G2 comprised those who had undergone chemotherapy, and G3 those who had undergone breast-conserving surgery and chemotherapy. Serum concentrations of retinol, β-carotene, and zinc were quantified. Breast cancer staging was based on the TNM (Tumor, Node, Metastasis) classification of malignant tumors, a type of staging tool for different cancers. Results: A total of 230 patients were assessed. A decrease of the serum concentrations of the micronutrients assessed as the staging level of the disease increased was observed. Surgery alone had a greater negative impact on serum concentrations of retinol. Considering the treatments prior to radiotherapy, patients undergoing surgery alone and chemotherapy associated with surgery had higher percentages of deficiency of β-carotene and retinol. There was a positive correlation between the concentrations of zinc, retinol, and β-carotene, showing a synergy between these micronutrients. Conclusion: A significant reduction in the serum concentrations of the assessed micronutrients was observed, according to the increase in breast cancer staging. The synergy between the micronutrients must be considered in order to maximize the benefits and minimize the adverse effects of irradiation to normal cells.

## 1. Introduction

Breast cancer is one of the most occurring malignant tumors in women. According to the Global Cancer Statistics (GLOBOCAN), worldwide, there will be about 2.1 million newly diagnosed female breast cancer cases in 2018, with 626,679 related deaths, accounting for almost 1 in 4 cancer cases among women [1].

Despite being considered a cancer with a relative favorable prognosis if diagnosed and treated properly, mortality rates for breast cancer are still high in Brazil [2]. However, survival rates are different when the disease is restricted to the organ where it started and when it spreads to other organs. Its classification in stages [3] is an important tool for evaluating the prognosis of these patients [4].

Cancer treatment aims to cure the disease, but when this is not possible, efforts focus on controlling the disease to extend life and preserve its quality. To achieve this goal, treatment protocols are proposed involving surgery, radiation therapy, chemotherapy, or a combination of these [5,6]. Treatments for this disease are able to generate free radicals, reduce intrinsic antioxidants, worsen pre-existing cancer comorbidities, or cause them to emerge as a result of the treatment itself [7].

Recently, the literature has been pointing out a relationship between nutrients with antioxidant function and cancer therapy. Among the antioxidants studied, some have been highlighted, such as vitamin A due to its role in cancer therapy, given its chemoprotective and antioxidant role, which is able to decrease, in the short and long term, the harmful effects of cancer treatment [8,9,10].

Zinc has an important antioxidant action, acts in the immune system and induces apoptosis in tumor cells [11]. In addition, this mineral is required for hepatic synthesis and secretion of retinol-binding proteins (RBP), and, in situations of deficiency, RBP synthesis may be impaired, resulting in a secondary vitamin A deficiency [12].

Despite the available knowledge, the use of these micronutrients in clinical practice is still controversial, and few scientific studies address this topic. In this context, the aim of this study is to investigate the relationship between serum concentrations of retinol, β-carotene, and zinc, according to the stage of breast cancer considering different treatment modalities prior to radiation therapy and the synergistic action between these micronutrients.

## 2. Methods

This is an observational and cross-sectional study in a cohort of free admission of patients with breast cancer prior to radiation therapy conducted in a private clinic in Rio de Janeiro state in the period from October 2011 to December 2012.

Patients were divided into 3 groups comprising those who had undergone breast-conserving surgery (G1)alone, chemotherapy alone(G2), and a combination of breast-conserving surgery and chemotherapy (G3). These treatment modalities were performed between 7 and 14 days prior to radiation therapy.

For biochemical analysis, an aliquot of blood was collected for dosage of vitamin A (retinol and β-carotene) and mineral zinc after at least 8 h of fasting. The samples were protected against oxidation and sent immediately to the laboratory to be analyzed.

The method for quantification of retinol and β-carotene used was high performance liquid chromatography by ultraviolet detector (HPLC-UV). The cut-off point used to detect retinol deficiency was <1.05 µmol/L and <50 µg/L to detect β-carotene [13]. The method used for quantification of zinc was atomic absorption spectrophotometry. The cut-off point used to indicate zinc deficiency was <587 mcg/L [14].

The study comprised women aged ≥20 years who had been diagnosed with breast cancer and its staging (TI-III N0 M0) based on the classification of malignant tumors proposed by the Union for International Cancer Control (UICC) [15], which was confirmed by histopathological reports and indication of external radiation therapy after breast-conserving surgery and/or chemotherapy.

Patients who showed disabsorptive syndromes, liver diseases, kidney diseases, diabetes mellitus, patients using, or who had used in the past 6 months a supplement containing vitamin A (retinol or β-carotene) or zinc were excluded from the study. Additionally, patients were excluded who had not had a regular clinical control after treatment, patients who died of a non-neoplastic disease-related death, and patients with missing data in their medical records.

Quantitative variables were expressed as mean and standard deviation, and qualitative variables were expressed as percentages. The Kolmogorov–Smirnov test was used to test the normality of continuous variables. The Mann–Whitney test was used to compare two groups, and the Friedman test was used to compare three groups. The Chi-square test was used to evaluate the association between categorical variables. Spearman’s correlation was used for correlation analysis. The significance level adopted was 5% (*p* < 0.05). The analyses were carried out with the Statistical Package for Social Sciences (SPSS) Program version 15.0.

This study was approved by the Ethics and Research Committee of Hospital Universitário Clementino Fraga Filho (CEP_HUCFF UFRJ 19109), and an informed consent form was signed by all the participants.

## 3. Results

A total of 230 women with mean age of 63.6 years (SD+9.3) were assessed. Age difference between the groups was not significant (*p* = 0.28) (Table 1).

With regards to the clinical stages of the disease, stage II predominated reaching 57.4% (n = 132) of the assessed patients. Stage I had 28.7% (n = 66) and stage III had 13.9% (n = 32) (Table 1).

Considering the studied groups, the highest percentage of retinol deficiency was in G3 reaching 21.3% (n = 27), and of β-carotene in G1 reaching 38.5% (20). The highest percentage of the mineral zinc was also found in G1 and reached 30.8% (n = 16) (Table 2).

When assessing the serum concentrations of antioxidants in the studied groups, significant differences were only observed in retinol (*p* < 0.01) (Table 3).

After assessing serum concentrations, it was observed that these antioxidants decreased as stages of breast cancer increased (Table 3).

When the comparison among the means of serum concentrations of retinol was performed according to the stages of the disease in the studied groups, it was observed that in G2 the differences were significant between stage I and III, and between stage II and III (both with *p* < 0.01 values). β-carotene showed statistical difference only when stage I was compared to stage III (*p* < 0.01). Zinc showed a behavior similar to retinol with statistical differences when stage I was compared to stage III and stage II to III (both with *p* < 0.01 values).

In G3, when the means of the micronutrients assessed in stage II were compared to stage III, it was observed that all showed statistically significant difference with *p* < 0.01 values.

With regard to comparison between the groups, it was observed that serum concentrations of retinol were significantly different between G1 and G2 (*p* = 0.03). However, the same did not occur with β-carotene (*p* = 0.26) and zinc (*p* = 0.06).

When assessing G1 and G3, no statistical difference was found in retinol and β-carotene (*p* = 0.29 and 0.07, respectively). However, statistical difference was found in zinc (*p* = 0.01).

When comparing G2 to G3, retinol was the only antioxidant nutrient that showed statistical difference (*p* < 0.01).

When analyzing the correlations between the assessed micronutrients, there was a positive correlation between retinol and β-carotene (*p* < 0.01 r = 0.358), between retinol and zinc (*p* < 0.01 r = 0.233), and between β-carotene and zinc (*p* = 0.03 r = 0.261).

## 4. Discussion

The results of the current study show that the more advanced stage of breast cancer, the smaller were the means of serum concentrations of retinol and β-carotene. Thus, our results corroborate the results of Matos et al. [16], who found a significant reduction in serum concentrations of vitamin A as breast cancer staging progressed. However, the percentage of β-carotene deficiency was different in G2 and G3, as no deficiency of this antioxidant in stage III was found in G3. This fact can be explained by an increase in the oxidative stress caused by the disease itself and made worse by the treatments received, which led to low-efficiency of β-carotene bioconversion to retinol [17], thus, causing an increase in retinol deficiency, as noted in our findings.

A similar behavior was observed in serum concentrations of zinc compared to retinol and β-carotene, i.e., the more advanced the stage of the disease, the greater the percentage of deficiency of this antioxidant nutrient. This finding corroborates the results of Kumar R. et al. (2017) [18], who found a significant decrease in serum concentrations of zinc as breast cancer staging progressed.

In our study, the highest prevalence of the disease was found in stage II, followed by stage I and III, with percentage values very close, corroborating the Instituto Nacional de Câncer(INCA) [National Institute of Cancer] national data (2012) [19] and the investigation carried out by Borges et al. (2013) [20], who found prevalence of stage II at the time of diagnosis. However, Soares et al. (2012) [21] reported a greater prevalence in stage III and IV at the time of diagnosis. It is noteworthy that the clinical stage of the disease is an important instrument for prognostic evaluation and that survival rates are related to the early detection of this disease [3].

The treatment of breast cancer is related to the clinical staging of the disease and depends on this classification. The procedures used are able to increase the production of free radicals and, consequently, to deplete antioxidants by the increase in oxidative stress [22]. In our study, among the treatments involved, the women who had only undergone surgery (G1) showed a more prominent reduction in serum concentrations of β-carotene and zinc. When assessing retinol, a reduction of serum concentrations in women who had undergone surgery and chemotherapy (G3) was observed, followed by the women who had only undergone surgery (G1), and no significant difference was observed between the groups. The trauma caused by surgery is associated with the increase in oxidative stress and excessive release of reactive oxygen species and reduction of antioxidant defenses [23]. Such findings demonstrate that the surgery alone, or in combination, had a negative impact similar to the one caused by the assessed antioxidants.

It is noteworthy that the women who had undergone surgery will still be exposed to radiation therapy, which may further increase the imbalance in the oxidant-antioxidant system, which is able to cause acute damage in the medium and long term, and may even result in secondary cancers [24]. In addition, free radicals can exacerbate the pre-existing comorbidities of cancer [25] and generate toxic effects such as cardiotoxicity, pulmonary, and renal toxicity [6].

In this context, retinol stands out for its antioxidant, proapoptotic, and anti-proliferative activity [25], and, as it reduces growth and cell division, it favors tumor cells death [26]. In addition, its adjuvant role in radiation therapy has been reported, making it more effective in the fight against the disease progression and in the reduction of side effects [27].

Carotenoids have antioxidant activity since they neutralize peroxyl radicals, in addition to singlet oxygen, thus reducing the oxidation of DNA and lipids. The mechanisms of action that contribute to their anticancer effects include antioxidant activity, inhibition of proliferation, effects on the immune system, and inhibition of the endogenous formation of carcinogens [28,29]. On the other hand, zinc has an important antioxidant action, has functions in metabolism and interacts with malignant cells, particularly with relation to apoptosis [30].

Although studies addressing these micronutrients in isolation have shown promising results in cancer therapy [25,26,29,30], studies have not yet considered the synergistic relationship between retinol, β-carotene, and zinc which, in addition to being potent antioxidants and closely linked to the immune system, have an important synergy.

Zinc is required for hepatic synthesis and secretion of retinol binding protein (RBP) and, in situations of deficiency, RBP synthesis may be impaired, resulting in secondary vitamin A deficiency [12]. According to Doldo et al. (2015) [25], in breast tumors a low regulation of RBP occurs, jeopardizing the transportation and the proapoptotic and anti-proliferative activity of retinol, thus, favoring tumor progression.

The low concentrations of zinc, often observed in patients with breast cancer who have undergone treatment protocols of this disease, generate an increase in oxidative stress [31,32,33], and may further worsen serum retinol deficiency as they cause a reduction in the liver mobilization of this vitamin [12]. Studies have reported that low concentrations of serum retinol are predictors of poor prognosis in patients with head and neck cancer [34] and hepatocellular carcinoma [35,36].

The synergy between the assessed micronutrients was observed in our study through the results that showed a positive correlation between retinol and β-carotene, between retinol and zinc, and between β-carotene and zinc. That is, as zinc deficiency occurred, the same occurred with respect to retinol, thus affecting serum concentrations of β-carotene. The better the status of retinol, the smaller will be the requirement of β-carotene to maintain its serum concentrations since this is the most powerful precursor of retinol [37]. This fact allows β-carotene to be more available in its antioxidant function, an action considered important in cancer control [29]. The results found in our study are in agreement with this finding, as the group with the lowest percentage of retinol deficiency (G2) also showed the lowest concentrations of β-carotene. However, we consider as a limitation of our study the impossibility of assessing other antioxidant micronutrients that could present synergy with the nutrients assessed in this study.

The findings presented here are important because, in addition to the role that these nutrients play in breast cancer treatment, they show that their joint use can promote the effectiveness of treatment by inhibiting the repair of the damage caused by irradiation in cancer cells, enhancing the expected damage caused by irradiation [26].

## 5. Conclusions

Our study showed a significant reduction in serum concentrations of retinol, β-carotene, and zinc, according to the increase in breast cancer staging. Considering the treatments prior to radiotherapy, patients undergoing surgery alone and chemotherapy associated with surgery had higher percentages of micronutrient deficiency, except for zinc in the group that only underwentchemotherapy.There was a positive correlation between deficiencies of zinc, retinol, and β-carotene, showing that the synergy between these micronutrients should be considered.

We recommend the follow-up of the nutritional status of the nutrients discussed in this study in conjunction with the different modalities of breast cancer treatments in order to maximize the benefits of treatment and minimize adverse effects to normal cells.

## Figures and Tables

**Table 1:** General characteristics of the sample.

Variables	Mean	SD	N
Age (years)			
G1	61.9	7.7	52
G2	64.6	9.2	51
G3	64.0	9.2	127
*p*-value	0.06		
Stage	I (%)	II (%)	III (%)
G1	100 (n = 52)	-	-
G2	27.4 (n = 14)	56.8 (n = 29)	15.6 (n = 8)
G3	-	81 (n = 103)	19 (n = 24)

Friedman and Kruskal–Wallis test. G1—women undergoing conservative mastectomy alone; G2—women undergoing chemotherapy alone; G3—women undergoing conservative surgery and chemotherapy.

**Table 2:** Percentage of antioxidant micronutrients deficiency according to groups that received treatments prior to radiation therapy and according to breast cancer staging.

Variables	Retinol (%)	β-Carotene (%)	Zinc (%)
G1	15.5 (n = 8)	38.5 (n = 20)	30.8 (n = 16)
G2	11.8 (n = 6)	7.8 (n = 4)	37.3 (n = 19)
G3	21.3 (n = 27)	10.2 (n = 13)	17.3 (n = 22)
G1			
Stage I	15.5 (n = 8)	38.5 (n = 20)	30.8 (n = 16)
Stage II	-	-	-
Stage III	-	-	-
G2			
Stage I	0	0	0
Stage II	10.3 (n = 3)	13.8 (n = 4)	37.9 (n = 11)
Stage III	37.5 (n = 3)	-	100 (n = 8)
G3			
Stage I	-	-	-
Stage II	17.5 (n = 18)	12.6 (n = 13)	42.7 (n = 44)
Stage III	37.0 (n = 9)	-	58.3 (n = 14)

Friedman and Kruskal–Wallis test. G1—women undergoing conservative mastectomy alone; G2—women undergoing chemotherapy alone; G3—women undergoing conservative surgery and chemotherapy.

**Table 3:** Serum concentrations of antioxidant micronutrients according to groups that received treatments prior to radiation therapy and according to breast cancer staging.

Variables	Retinol (µmol/L)	β-Carotene (µg/L)	Zinc (mcg/L)
G1	1.56 ± 0.073	171.11 ± 180.2	671.8 ± 170.2
G2	1.87 ± 0.072	203.6 ± 101.5	750.4 ± 249.8
G3	1.46 ± 0.050	227.1 ± 157.8	752.2 ± 193.6
*p*-value	<0.01	0.083	0.045
G1			
Stage I	1.57 ± 0.74	171.1 ± 180.2	671.6
Stage II	-	-	-
Stage III	-	-	-
G2			
Stage I	2.15 ± 0.42	245.3 ± 29.0	728.6 ± 14.1
Stage II	1.99 ± 0.78	196.7 ± 128.2	840.10 ± 278.8
Stage III	1.02 ± 0.18	156.0 ± 0.1	460.4 ± 54.7
*p*-value	<0.01	0.11	<0.01
G3			
Stage I	-	-	-
Stage II	1.56 ± 0.50	247.1 ± 166.3	823.7 ± 133.6
Stage III	1.04 ± 0.23	141.1 ± 64.5	500.9 ± 172.3
*p*-value	<0.01	<0.01	<0.01

Friedman and Kruskal–Wallis test. G1—women undergoing conservative mastectomy alone; G2—women undergoing chemotherapy alone; G3—women undergoing conservative surgery and chemotherapy.
